# Impact of 9p deletion and p16, Cyclin D1, and Myc hyperexpression on the outcome of anaplastic oligodendrogliomas

**DOI:** 10.1371/journal.pone.0193213

**Published:** 2018-02-28

**Authors:** Karine Michaud, Marie de Tayrac, Myreille D’Astous, Claudie Paquet, Peter Vincent Gould, Stéphan Saikali

**Affiliations:** 1 Department of Neurosurgery, Centre Hospitalier Universitaire de Québec, Québec, Canada; 2 Department of Genomic and Molecular Genetics, Centre Hospitalier Universitaire de Rennes, Rennes, France; 3 Department of Pathology, Centre Hospitalier Universitaire de Québec, Québec, Canada; Seconda Universita degli Studi di Napoli, ITALY

## Abstract

**Objective:**

To study the presence of 9p deletion and p16, cyclin D1 and Myc expression and their respective diagnostic and prognostic interest in oligodendrogliomas.

**Methods:**

We analyzed a retrospective series of 40 consecutive anaplastic oligodendrogliomas (OIII) from a single institution and compared them to a control series of 10 low grade oligodendrogliomas (OII). Automated FISH analysis of chromosome 9p status and immunohistochemistry for p16, cyclin D1 and Myc was performed for all cases and correlated with clinical and histological data, event free survival (EFS) and overall survival (OS).

**Results:**

Chromosome 9p deletion was observed in 55% of OIII (22/40) but not in OII. Deletion was highly correlated to EFS (median = 29 versus 53 months, p<0.0001) and OS (median = 48 versus 83 months, p<0.0001) in both the total cohort and the OIII population. In 9p non-deleted oligodendrogliomas, p16 hyperexpression correlated with a shorter OS (p = 0.02 in OII and p = 0.0001 in OIII) whereas lack of p16 expression was correlated to a shorter EFS and OS in 9p deleted OIII (p = 0.001 and p = 0.0002 respectively). Expression of Cyclin D1 was significantly higher in OIII (median expression 45% versus 14% for OII, p = 0.0006) and was correlated with MIB-1 expression (p<0.0001), vascular proliferation (p = 0.002), tumor necrosis (p = 0.04) and a shorter EFS in the total cohort (p = 0.05). Hyperexpression of Myc was correlated to grade (median expression 27% in OII versus 35% in OIII, p = 0.03), and to a shorter EFS in 9p non-deleted OIII (p = 0.01).

**Conclusion:**

Chromosome 9p deletion identifies a subset of OIII with significantly worse prognosis. The combination of 9p status and p16 expression level identifies two distinct OIII populations with divergent prognosis. Hyperexpression of Bcl1 and Myc appears highly linked to anaplasia but the prognostic value is unclear and should be investigated further.

## Introduction

The major aim of this study was to continue our previous work [1] on the prognostic value of chromosome 9p status in anaplastic oligodendrogliomas (OIII) and to confirm the reliability of the FISH technique using a standard FISH platform, an easily available commercial probe and an automated software analysis package with a previously established algorithm [2].

Since 2016, the WHO defines oligodendrogliomas (OGs) by the molecular genetic features of 1p/19q whole arm codeletion and IDH1/2 mutation [3,4]. These tumors are sensitive to chemotherapy given alone or after radiotherapy, with a global favorable outcome [5,6]. Additional genetic aberrations have been associated with higher grade OGs, in particular 9p loss, 9q loss, 10q loss, 11q gain, whole chromosome 7 gain and whole chromosome 4 loss [7,8]. Recent studies underlined the prognostic value of 9p deletion in OGs, which appears linked to two of the major histologic criteria of anaplasia traditionally used to define OIII, namely microvascular proliferation (MVP) and tumor necrosis [8–10] and may provide a genetic explanation for tumor progression in these cases [9].

In our previous study we showed the feasibility and reliability of an automated FISH technique for the study of chromosome 9p status in oligodendroglial tumors [1] but our conclusions were limited by the small size of the OG cohort. In the present study we wanted to confirm our previous findings on a larger cohort of well-defined IDH mutated and 1p/19q codeleted OG. At the same time we also wished to evaluate protein p16 (CDKN2A) expression in this cohort as a possible diagnostic and /or prognostic marker since the *CDKN2A* gene is located on 9p21. Finally, we studied the diagnostic and prognostic value of two additional proteins which have recently been implicated as markers of anaplasia and short outcome in OG [11,12] and which are also linked to p16: Cyclin-D1 (CCND1) which dimerizes with CDK4, the main target of p16 [13] and Myc (c-Myc) which impacts a wide number of cellular processes and may influence p16 via overexpression of HGMA2 and downregulation of CDKN2A [14].

## Materials and methods

### Ethics statement

The local Institutional Care and Use Committee (IACUC) (ethics committee) of the Centre Hospitalier Universitaire de Québec was consulted and approved this study (notice 2017–3456): S1 File. Tumor samples were collected and anonymized by the Pathology Service of the Centre Hospitalier Universitaire de Québec (Hôpital de l’Enfant-Jésus, Quebec City, Canada).

### Patients and tissue specimens

Formalin fixed paraffin-embedded (FFPE) tissue from 40 consecutive brain OIII samples (biopsies or surgical resections) studied in our institution between 1998 and 2015 were selected for this study. An additional sampling from a consecutive cohort of 10 OII managed at the same period (2000 to 2004) was selected as a control. The OIII series was assembled from all high grade gliomas diagnosed during this period and presenting an oligodendroglial component and/or a 1p/19q codeletion. All selected cases were analyzed by two neuropathologists (PVG and SS) and reclassified according to the recent WHO 2016 guidelines [3]. Original diagnoses for this cohort included 32 OIII, 2 anaplastic astrocytomas (AIII), 5 anaplastic oligoastrocytomas (OAIII) and one glioblastoma rich in oligodendroglioma (GBMO).

### Immunohistochemistry

Immunohistochemistry (IHC) was performed on 4 μm thick FFPE sections with the EnVision^™^ FLEX+ detection system on Dako Autostainer 48 (Dako, Mississauga, Ontario). Reaction was visualized by EnVision^™^ FLEX DAB+ Chromogen (Dako, Glostrup, Denmark).

The cases were screened using monoclonal antibodies IDH1 R132H (clone H09; dilution 1/50; Optistain), Internexin Alpha (INA; clone 2E3; dilution 1/200; Santa Cruz), ATRX (clone D-5; dilution 1/50; Santa Cruz), p53 (clone DO-7; no dilution; Dako), Ki67 (clone MIB1; no dilution; Dako), p16 (clone E6H4; dilution 1/6; Roche), Cyclin D1/Bcl1 (clone SP4; no dilution; Dako), and c-Myc (clone EP121; dilution 1/100; Cell Marque) with an incubation time of 30 min for each.

MIB1, p53, p16, Cyclin D1 and c-Myc expression was scored as a percentage by counting the immunostained nuclei of 100 tumor cells in the most positive area. p53 staining was considered positive when ≥ 50% of tumor cells presented a strong nuclear staining. INA expression was scored as positive when ≥ 10% of cells were positive with at least one cluster of positive cells [15]. IDH R132H expression was scored as positive (mutated) if at least one positive cell was observed or negative (wild type). ATRX expression was scored as positive (wild type) if ≥ 10% of cells were positive or negative (mutated) [16]. p16 and Cyclin D1 expression was scored as negative if less than 7% or 6% of cells respectively were positive which corresponds to the mean expression + 2SD of the normal brain control cohort.

### FISH technique

FISH analysis of 9p status was performed using the Vysis CDKN2A/CEP 9 FISH Probe Kit (LSI 9p21 CDKN2A/9p11-q11 CEP 9; Abbott Molecular Inc., Abbott Park, Illinois, USA). Briefly, 5-μm-thick formalin-fixed, paraffin-embedded sections were deparaffinized, treated with saline sodium citrate and digested in pepsin solution. The probe mixture was prepared according to the manufacturer’s instructions and an appropriate volume was added to each slide. Target DNA and probes were codenatured at 74°C for 5 minutes and incubated at 37°C overnight in a humidified hybridization chamber (Thermo-Brite, Abbott Molecular Inc.). Post-hybridization washes were performed in NP40 0.3%/2×SSC (pH 7.0) at 75°C for 2 minutes. Finally, the slides were air dried and counterstained with DAPI (40,6-diamidino-2-phenylindole) diluted in Vectashield (Vector, Burlingame, CA, USA). Signal acquisition was performed for each slide over 12 more representative areas. These areas were automatically captured at x400 using a Metasystem station (Zeiss MetaSystems, Thornwood, NY) equipped with a Zeiss Axioplan fluorescent microscope. The acquired images were then used as the basis for automated analysis performed by the Metafer 4 software package (Metasystems).

### FISH interpretation

9p status FISH analysis was automatically performed on all tumor cells identified by the Metafer 4 software using our internal algorithm [1,2]. This sampled an average of 891 cells (min: 90 –max: 1377 –median: 918) for chromosome 9p in the whole series.

The deletion status cut-off was calculated on a series of 5 normal autopsy brains using mean+2SD and was set at 20%. A tumor was classified as deleted if the percentage of deleted nuclei exceeded the deletion status cut-off of 20%. Otherwise a tumor was classified as non-deleted.

### Statistical analyses

Statistical analyses was performed using the R statistical environment (http://www.R-project.org/). Chi-square tests were used for group comparisons between clinical, histological and molecular status data. The strength of the linear relationship between quantitative variables was assessed using correlation analysis. Survival curves were obtained according to the Kaplan-Meier method and compared using the log-rank test. Event Free Survival (EFS) was defined as the time from diagnosis to the first recurrence. Overall Survival (OS) was defined as the time from diagnosis to death or last follow-up. The cohort was followed from 1998/01/01 to 2017/06/01. The following variables were queried for prognostic significance across the whole group: histological grade, recurrence (yes or no), age at diagnosis (cut off = 50 years), sex, extent of surgery (biopsy versus surgery), postoperative treatment (radio/chemotherapy versus none), microvascular proliferation (MVP) (present/absent), necrosis (present/absent), calcifications (present/absent), number of mitoses (cut off = 5 mitosis /10 HPF, median), INA expression (cut off = 10%), MIB1, p16, Cyclin D1 and Myc labelling index and 9p deletion. Variables that were significant in univariate analysis were evaluated in a multivariate cox regression model. Values ≤ 0.05 were considered statistically significant.

## Results

### Clinical data

Clinical data are summarized in Table 1. Our series of 50 patients included 28 males (56%) and 22 females (44%). The cohort’s mean age at diagnosis was 48 years. Patients underwent open surgery with gross total or subtotal tumor resection in 47 cases (94%) and stereotactic biopsies in 3 cases (6%). The majority of cases were located in the frontal lobe (39/50 = 78%) followed by the temporal lobe (8/50 = 16%), the parietal lobe (2/50 = 4%) and the occipital lobe (1/50 = 2%). Twelve cases were recurrent tumors (12/50 = 24%). Ten patients had no post-operative treatment (20%), 3 patients received post-operative radiotherapy alone with a mean total dose of 50Gy (6%), 25 patients received chemotherapy alone with PCV or Temozolomide (50%) and 12 patients (24%) were treated with adjuvant radio-chemotherapy (PCV or Temozolomide).

**Table 1:** Clinical, histological and molecular data according to the tumoral grade and 9p deletion status.

Histological Type		Total cohort	OII	OIIIw	OIIId	OII/ OIII	OIIIw/OIIId	OII+OIIIw/OIIId
**N**		50	10	18	22	10/40	18/22	28/22
**Recurrence**	**Yes (%)**	12(24)	0(0)	4 (22)	8 (36)	**0.0002**	NS	0.08
**Age at diagnosis**	**Mean**	48	45	44	51	NS	**0.03**	**0.04**
	**Median**	46	41	44	49			
**Sex**	**Male (%)**	28 (56)	6 (60)	7 (39)	15 (68)	NS	NS	NS
	**Female (%)**	22 (44)	4 (40)	11 (61)	7 (32)			
**Extent of surgery**	**Biopsy (%)**	3 (6)	1 (10)	1 (6)	1 (5)	NS	NS	NS
	**Surgery (%)**	47 (94)	9 (90)	17 (94)	21 (95)			
**Localization**	**Frontal (%)**	39 (78)	9 (90)	15 (83)	15 (68)	NS	NS	NS
	**Temporal (%)**	8 (16)	1 (10)	2 (11)	5 (24)			
	**Parietal (%)**	2 (4)	0 (0)	1 (6)	1 (4)			
	**Occipital (%)**	1 (2)	0 (0)	0 (0)	1 (4)			
**Postoperative treatment (%)**	**None**	10 (20)	4 (40)	2 (12)	4 (18)	**0.008**	NS	NS
	**Chemotherapy**	25 (50)	5 (50)	8 (44)	12 (55)			
	**Radiotherapy**	3 (6)	1 (10)	0 (0)	2 (9)			
	**Radio + chemotherapy**	12 (24)	0 (0)	8 (44)	4 (18)			
**Status**	**Dead (%)**	28 (56)	7 (70)	5 (28)	16 (73)	NS	**0.004**	**0.03**
**MVP**	**Glomeruloid (%)**	40 (45)	0 (0)	18 (100)	22 (100)	**-**	**-**	**0.0006**
**Necrosis**	**Present (%)**	22 (44)	0 (0)	7 (39)	15 (68)	**<0,0001**	0.07	**0.002**
**Calcifications**	**Present (%)**	23 (46)	4 (40)	9 (50)	10 (45)	NS	NS	NS
**Mitoses / 10 HPF**	**Mean**	8	2	11	8	**<0,0001**	NS	NS
	**Median**	5	2	6	5			
**MIB1 (%)**	**Mean**	22	9	29	23	**<0,0001**	0.09	NS
	**Median**	22	10	26	24			
**INA**	**Positive (%)**	38 (76)	8 (80)	15 (83)	15 (68)	NS	NS	NS
**IDH1/2 mutation**	**Presence (%)**	50 (100)	10 (100)	18 (100)	22 (100)	NS	NS	NS
**ATRX mutation**	**Absence (%)**	50 (100)	10 (100)	18 (100)	22 (100)	NS	NS	NS
**p16 (%)**	**Mean**	17	13	20	17	0.07	NS	NS
	**Median**	12	12	14	12			
**Cyclin D1 (%)**	**Mean**	37	20	41	42	**0.0006**	NS	NS
	**Median**	38	14	43	45			
**Myc (%)**	**Mean**	35	26	41	34	**0.03**	NS	NS
	**Median**	33	27	34	35			
**Chr 9p**	**Loss (%)**	22 (44)	0 (0)	0 (0)	22 (100)	**<0,0001**	**-**	**-**

Statistically significant: p-values <0.05, NS: not significant,—: Not applicable, MVP: microvascular proliferation, INA: alpha-internexin, Chr: chromosome, HPF: high power-field, OII: grade II oligodendroglioma, OIIIw: anaplastic oligodendroglioma 9p wild type, OIIId: anaplastic oligodendroglioma 9p deleted

The cohort mean EFS was 52 months (min = 1; max = 200, median = 40 months) and mean OS was 76 months (min = 6; max = 211, median = 61 months). 28 patients were dead at the end of the study (56%).

### Histological data

The cohort was categorized into 3 subgroups according to WHO 2016 grading and chromosome 9p deletion status: OII, OIII - 9p wild type (OIIIw) and OIII with 9p deletion (OIIId). No significant difference was observed between the sex, the localization and the extent of surgery distribution according to the 3 subgroups (Table 1). The mean and median age at diagnosis for the OIIIw subgroup was significantly younger than the OIIId subgroup (p = 0.03). A significant proportion of OII patients was not treated by radiotherapy and/or chemotherapy compared to those of OIIIw and OIIId subgroups (p = 0.008).

MVP, high mitotic index (≥5/10 HPF) and necrosis were linked to OIII subgroups (p = 0.0006, p<0.0001 and p<0.0001 respectively) as defined by the WHO 2016 classification without significant difference between OIIIw and OIIId subgroups.

Calcifications was not correlated to any subgroup.

### Immunohistochemistry

**Fig 1:**
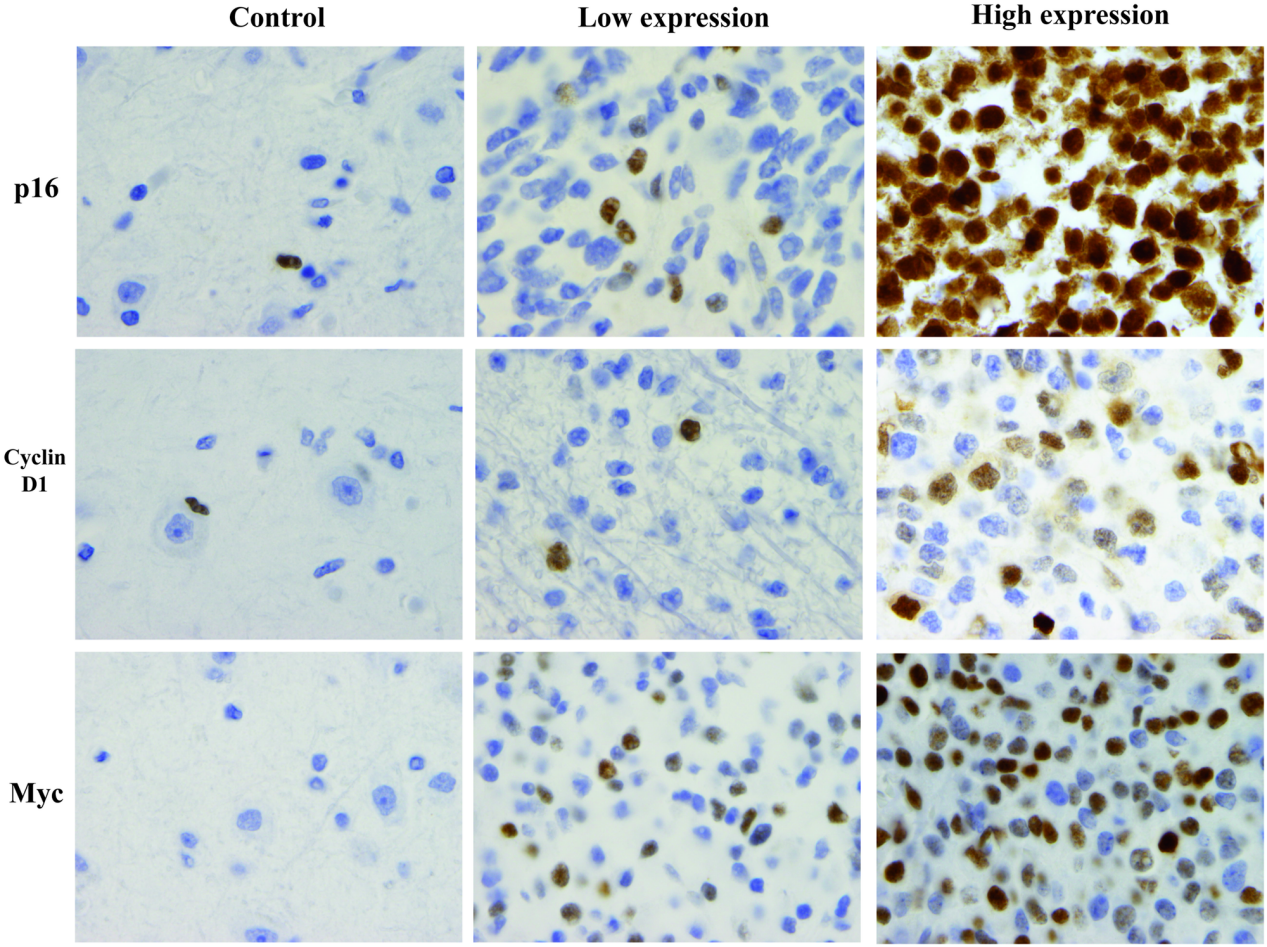
Representative low and high nuclear immunostaining for p16, Cyclin D1 and Myc and their normal brain control. For p16 and Cyclin D1 rare astrocytes are positive in normal cortex whereas no staining is observed for Myc (magnification x 1000 for all pictures).

For p16, Cyclin D1 and Myc, a strong nuclear staining was observed in some tumor cells in all cases, which allowed for the easy calculation of positive cell percentage for each case (Fig 1).

**Fig 2:**
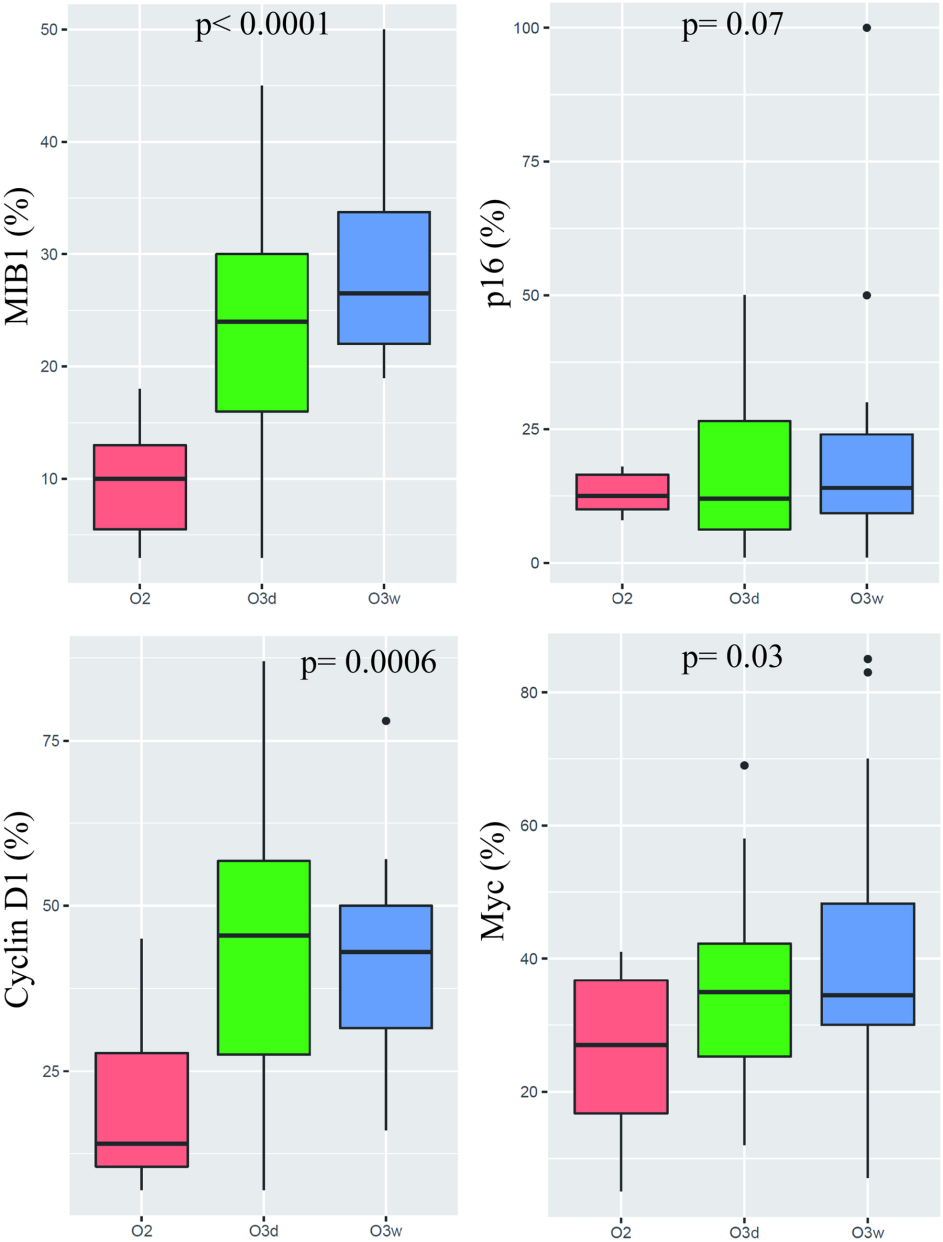
Boxplots of nuclear percentage of expression for MIB1, p16, Cyclin D1, and Myc according to the histological type. O2: Low grade oligodendroglioma. O3d: 9p deleted anaplastic oligodendroglioma. O3w: 9p wild type anaplastic oligodendroglioma.

MIB1 expression was present in both OII and OIII (mean = 9% and >23% respectively). Overexpression of MIB1 was highly correlated to the histological grade (Fig 2) but not to chromosome 9 deletion (p<0.0001 and p = 0.09 respectively): Table 1.

INA expression was present in the majority of the cohort (76%) with no significant correlation to the grade or the chromosome 9 deletion (Table 1).

IDH1 R132H expression which is correlated to *IDH1* gene mutation was present in the majority of OII (9/10 = 90%) and OIII (38/40 = 95%). The three IDH1 R132H negative cases were mutated for IDH2 on complementary molecular analysis (Table 1).

Loss of ATRX protein expression which is correlated to *ATRX* gene mutation [16] was not observed in any of our cases (Table 1).

The majority of our cases showed an overexpression of these proteins with a mean expression of 17% for p16 (min = 1%; max = 100%, median = 12%), 37% for Cyclin D1 (min = 7%; max = 87%, median = 38%) and 35% for Myc (min = 5%; max = 85%, median = 33%) compared to normal brain control (mean = 3% for p16, 2% for Cyclin D1 and 0% for Myc respectively). No significant difference in p16 expression was observed between the OII and OIII cases (median = 12% versus 13%, mean = 12% versus 18%, p = 0.07): Fig 2. Cyclin D1 overexpression was significantly higher in OIII compared to OII (median = 45% versus 14%, p = 0.0006): Fig 2. Myc overexpression was also significantly higher in OIII compared to OII (median = 35% versus 26%, p = 0.03): Fig 2.

No significant difference of expression attributable to 9p status was observed for either proteins (Table 1). In our study, all cases overexpressed Cyclin D1 or Myc compared to normal brain controls. On the other hand we observed an absence of p16 expression in 11/40 OIII (27%) cases with no significant difference attributable to 9p status: 22% in OIIIw (4/18) and 32% (7/22) in OIIId respectively.

### Molecular data

Automated analysis of 9p was easily performed in all the cases without need for a manual control. None of the OII cases presented a 9p deletion. In the OIII cohort, 22 cases were deleted for 9p (22/40 = 55%). The correlation of 9p deletion to the histological grade appeared very strong in univariate and multivariate analysis (p<0.0001). 9p deletion was also strongly correlated with the presence of tumor necrosis (p = 0.003) but not to the presence of MVP (Table 2).

**Table 2:** Correlation between immunohistochemistry, histological data and 9p deletion: Univariate and multivariate analysis.

	MIB1	INA	p16	Cyclin D1	Myc	Chr 9p del
**Histological Type**	<0,0001*	NS	NS	0.002*	0.06	<0,0001*
**MVP**	**<0,0001**	NS	NS	**0.002**	0.06	NS
**Necrosis**	NS	**0.01**	NS	**0.04**	NS	**0.003**
**MIB1 (%)**	/	NS	NS	<0,0001*	NS	NS
**INA status**	NS	/	NS	NS	NS	NS
**p16 (%)**	NS	NS	**/**	NS	**0.01**	NS
**Cyclin D1 (%)**	<0,0001*	NS	NS	/	NS	NS
**Myc (%)**	NS	NS	**0.01**	NS	/	NS
**Chr 9p del**	NS	NS	NS	NS	NS	/

Statistically z test significant: p-values <0.05, NS: not significant, /: Not applicable, MVP: microvascular proliferation, INA: alpha-internexin, Chr 9p del: chromosome 9p deletion,

*: also significant in multivariate analysis.

### Correlation between clinical, histological and molecular data

In this study, OIII cohort was significantly correlated with a higher expression of both MIB1 (p<0.0001) and Cyclin D1 (p = 0.002) in both univariate and multivariate analysis (Table 2). The presence of MVP also correlated with MIB1 and Cyclin D1 overexpression in univariate analysis (p<0.0001 and p = 0.002 respectively). Myc overexpression showed a trend towards significant correlation with OIII grade and presence of MVP in univariate analysis (p = 0.06 for both data). Tumor necrosis appeared correlated to INA expression and Cyclin D1 overexpression (p = 0.01 and p = 0.04 respectively). MIB1 and Cyclin D1 overexpression were significantly correlated (p<0.0001) in both univariate and multivariate analysis. Myc and p16 overexpression were correlated in univariate analysis (p = 0.01). In the OIIId subgroup, absence of p16 expression was correlated with higher MIB1 expression (mean MIB1 = median = 30% in p16 negative cases versus mean MIB1 = median = 20% in p16 positive cases; p = 0.02) but not to Cyclin D1 overexpression (45% versus 45%, p = NS). On the other hand in the OIIIw subgroup it was the p16 overexpression (>30%) which correlated with MIB1 overexpression (Median MIB1 = 45% versus 25%; p = 0.04) and Cyclin D1 overexpression (median = 50% versus 40%, p = 0.04). In the OIIIw subgroup, absence of p16 expression showed no correlation with Cyclin D1 or MIB1 overexpression (median = 44% versus 40% and median = 30% versus 29% respectively; p = NS). In OII, p16 overexpression showed no correlation with MIB1 or Cyclin D1 overexpression (median = 10% versus 10% and median = 12% versus 14% respectively; p = NS).

### Correlation of clinical, histological and molecular data with EFS and OS

In our series, histological grading showed no correlation with EFS or OS (Table 3) despite a higher median EFS and OS in OII compared to OIII (62 months versus 33 months and 110 months versus 53 months respectively).

**Table 3:** Correlations between clinical, histological and molecular data and survival: Univariate and multivariate analysis.

		Total cohort		OII		OIII		OIIIw		OIIId	
		EFS	OS	EFS	OS	EFS	OS	EFS	OS	EFS	OS
Histological grade	Low / High grade	NS	NS	/	/	/	/	/	/	/	/
Recurrence	No / Yes	0.0003*	<0.0001*	/	/	**0.002**	**<0.0001**	NS	**0.01**	**0.001**	**0.0001**
Age at diagnosis	< 50 / ≥ 50 years	NS	NS	NS	NS	NS	NS	NS	NS	NS	NS
Sex	Male / Female	NS	NS	NS	NS	NS	NS	NS	NS	NS	NS
Extent of surgery	Biopsy / Surgery	NS	NS	NS	0.003	NS	NS	NS	NS	NS	NS
Localization	Frontal / Other	**0.004**	**0.05**	NS	NS	**0.04**	NS	NS	NS	NS	NS
Postoperative treatment	No / Yes	NS	NS	NS	NS	NS	NS	NS	NS	NS	NS
MVP	No / Yes	NS	NS	/	/	/	/	/	/	/	/
Necrosis	No / Yes	**0.03**	**0.001**	/	/	0.07	**0.01**	NS	NS	NS	NS
Calcifications	No / Yes	NS	**0.03**	NS	NS	NS	**0.04**	NS	NS	**0.05**	**0.04**
Mitoses /10 HPF	< 5 / ≥ 5	**0.02**	**0.0004**	NS	NS	0.06	**0.003**	**0.01**	**0.001**	**0.008**	**0.001**
MIB1	< 20 / ≥ 20%	**0.04**	**0.003**	NS	NS	**0.04**	**0.004**	**0.02**	**0.002**	**0.002**	**<0.0001**
INA	Negative / Positive	NS	NS	NS	NS	NS	NS	NS	NS	NS	NS
p16	< 12 / ≥ 12%	NS	NS	NS	**0.02**	NS	NS	NS	NS	NS	NS
p16	Expression Yes / No	NS	NS	/	/	NS	NS	NS	NS	**0.001**	**0.002**
Cycline D1	< 37 / ≥ 37%	**0.05**	0.09	NS	NS	0.08	NS	NS	NS	NS	NS
Myc	< 35 / ≥ 35%	NS	NS	NS	NS	NS	NS	**0.01**	NS	NS	NS
Chr 9p	Deletion / None	<0.0001*	<0.0001*	/	/	**0.0004**	**0.001**	/	/	/	/

EFS: Event Free Survival, OS: Overall Survival, NS: Not significant, /: Not applicable,

*: also significant in multivariate analysis.

**Fig 3:**
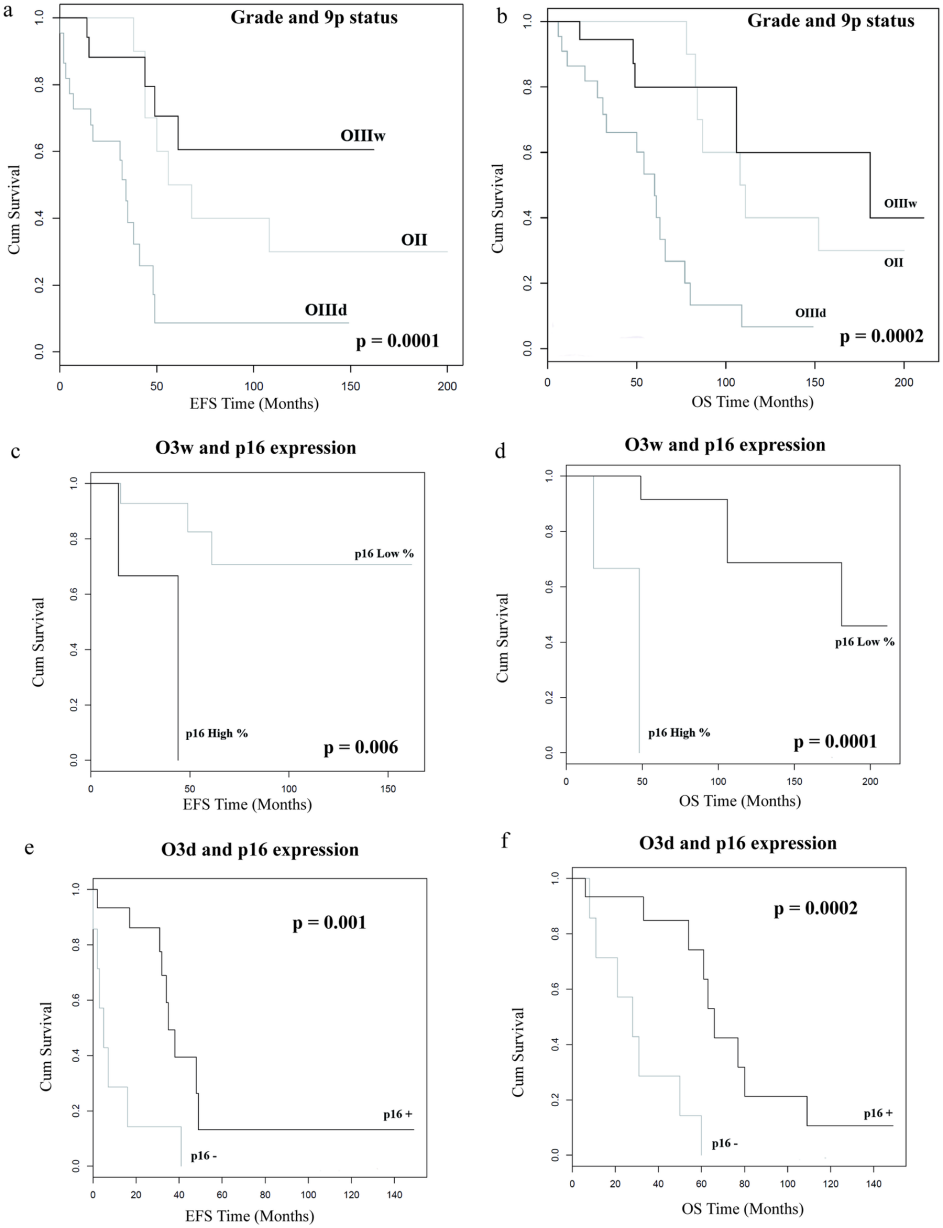
Event Free Survival (EFS) and Overall Survival (OS) according to the histological grade and the 9p deletion status. 9p deleted anaplastic oligodendrogliomas (OIIId) are associated with shorter EFS and OS than non-deleted anaplastic oligodendrogliomas (OIIIw) and low grade oligodendrogliomas (OII) (a and b). Interpretation of p16 expression is radically different according to the 9p status in anaplastic oligodendrogliomas (OIII): hyperexpression of p16 (>30%) is correlated to shorter EFS and OS in non-deleted OIII (c and d) whereas absence of expression of p16 (<7%) is correlated to shorter EFS and OS in deleted OIII (e and f).

Chromosome arm 9p deletion appeared strongly linked to a poor survival in the total population for both EFS (median = 29 versus 53 months, p<0.0001) and OS (median = 48 versus 83 months, p<0.0001). This poor prognosis remained statistically significant in the OIII cohort. The OIIId subgroup had a shorter EFS (median = 29 versus 46 months, p = 0.0004) and OS (median = 48 versus 61 months, p = 0.001) than the OIIIw (Table 3) or OII subgroups (Fig 3a and 3b). EFS median was 29 months for OIIId, 46 months for OIIIw and 62 months for O2 respectively (p = 0.0001). OS median was 48 months for OIIId, 61 months for OIIIw and 109 months for O2 respectively (p = 0.002).

Glioma recurrence was not observed in the OII subgroup. In the OIII subgroup recurrence was highly correlated with a shorter EFS (median = 16 versus 29 months, p = 0.002) and OS (median = 40 versus 61 months, p<0.0001), particularly in OIIId subgroup (p = 0.001 for EFS and p = 0.0001 for OS): Table 3.

Age at diagnosis over 50 years and sex were not correlated with shorter EFS or OS in our cohort. Surgical resection was correlated with a better OS than biopsy alone in the OII subgroup (median = 111 versus 78 months, p = 0.003). Frontal localization was correlated to a longer EFS (median = 47 versus 32 months, p = 0.004) and OS (median = 66 versus 54 months, p = 0.05) in the overall cohort.

Presence of tumor necrosis correlated with a shorter EFS (median = 23 versus 47 months, p = 0.03) and OS (median = 49 versus 80 months, p = 0.001) in the total cohort and with a shorter OS in the combined OIII cohort (median = 49 versus 64 months, p = 0.01). Presence of calcifications correlated with a higher OS in the series as a whole (median = 66 versus 54 months, p = 0.03), the combined OIII cohort (median = 61 versus 49 months, p = 0.04) and the OIIId subgroup (median = 62 versus 32 months, p = 0.04). In this subgroup, presence of calcifications was also correlated to EFS (median = 36 versus 16 months, p = 0.05): Table 3.

High mitotic index (cut off > 5 mitosis /10HPF) correlated with a shorter EFS (median = 31 versus 48 months, p = 0.02) and OS (median = 49 versus 79 months, p = 0.0004) in the cohort as a whole. In the combined OIII cohort it correlated with a shorter OS (median = 48 versus 59 months, p = 0.003). It also correlated with a shorter EFS (median = 41 versus 59 months, p = 0.01) and OS (median = 50 versus 63 months, p = 0.001) in the OIIIw subgroup and with a shorter EFS (median = 16 versus 34 months, p = 0.008) and OS (median = 31 versus 54 months, p = 0.001) in the OIIId subgroup: Table 3.

The MIB1 labelling index presented a similar correlation with EFS and OS to the mitotic index. MIB1 high proliferative index (cut off > 20%) correlated with a shorter EFS (median = 30 versus 45 months, p = 0.04) and OS (median = 49 versus 81 months, p = 0.003) in the cohort as a whole. In the combined OIII cohort it correlated with a shorter EFS (median = 30 versus 36 months, p = 0.04) and OS (median = 49 versus 59 months, p = 0.004). It also correlated with a shorter EFS and OS in the both OIIIw cohort (median EFS = 44 versus 57 months, p = 0.02, median OS = 49 versus 61 months, p = 0.002) and the OIIId cohort (median EFS = 7 versus 32 months, p = 0.002, median OS = 33 versus 52 months, p<0.0001): Table 3.

INA overexpression showed no prognostic significance in our series (Table 3).

In the cohort as a whole p16 overexpression (cut off = 12%) was not correlated with EFS or OS (Table 3). In the OII cohort p16 overexpression was significantly correlated with a shorter OS (median = 84 versus 152 months, p = 0.02). There was a trend towards shorter EFS without statistical significance (median = 56 versus 108 months, p = 0.2). More detailed statistical analysis of the OIII cohort revealed two distinct p16 expression profiles which varied in impact according to chromosome 9p status. In the OIIIw subgroup a very high p16 overexpression (p16 > 30%, which corresponds to the highest quartile of this cohort) correlated with shorter EFS (median = 29 versus 57 months, p = 0.006) and OS (median = 32 versus 66 months, p = 0.0001): Fig 3c and 3d respectively. At the opposite extreme in the OIIId subgroup, the absence of expression of p16 (p<7%) correlated with shorter EFS (median = 5 versus 32 months, p = 0.001) and OS (median = 28 versus 54 months, p = 0.0002): Fig 3e and 3f respectively.

Cyclin D1 overexpression (cut off = 37%) was correlated with shorter EFS in the cohort as a whole (median = 35 versus 44 months, p = 0.05). Overexpression also showed a trend towards shorter OS without statistical significance (median = 61 versus 66 months, p = 0.09). Within the OIII series, Cyclin D1 overexpression was not correlated with EFS or OS (Table 3) for either OIIIw or OIIId.

A prognostic impact of Myc expression was only observed in the OIIIw subgroup where overexpression of Myc (cut off = 35%) was correlated with shorter EFS (median = 39 versus 66 months, p = 0.01).

In a multivariate analysis of clinical, histological and molecular data, only chromosome 9p deletion and tumor recurrence remained predictive of poor prognosis associating with both shorter EFS (p = 0.0001 and p = 0.001 respectively) and OS (p = 0.0005 and p<0.0001 respectively).

## Discussion

As per the WHO 2016 classification of tumors of the central nervous system [3,4], our series of OG showed the double molecular signature of IDH mutation and 1p/19q codeletion. As expected, the majority of our 50 cases showed a classical oligodendroglial morphology with monomorphic cells, uniform round nuclei and perinuclear haloes and classical honeycomb architecture. Only 8 cases (16% of the cohort), previously classified as astrocytomas or mixed oligoastrocytomas, were been reclassified into OG as a result of their molecular signature. This degree of reclassification is in concordance with the literature [17].

In our series, men were affected more frequently than women with a ratio of 1.3:1 and the median age at diagnosis was higher in OIIIs than OIIs (47 versus 41 years) similar to previous literature [18]. As expected, the majority of OGs were located in the frontal lobe (nearly 80%) then, in order of decreasing frequency, in the temporal, parietal and occipital lobes [4,19]. Frontal lobe localization was correlated to a longer EFS and OS [19]. There was no difference between the OII and OIII subgroups regarding other parameters except for postoperative treatment, with a higher percentage of untreated patients in the OII cohort than in the OIII cohort (40% versus 15%). Excluding tumor localization, these clinical parameters did not correlate with prognosis in this study, similar to previous literature [8].

According to the WHO 2016 criteria, high mitotic index, presence of MVP and tumor necrosis distinguish OIII from OII. In our series necrosis and high mitotic and MIB1 proliferative index had a strong negative predictive value, similar to previous literature [20]. A high proliferative index correlated with shorter EFS and OS within the OIII cohort, similar to previous literature [21]. Intratumoral calcifications were observed in approximatively half of our cases (46%) without discrimination between OII and OIII, similar to previous literature [4,20]. These calcifications correlated with a better OS in univariate analysis, as reported in few studies [22].

In addition to the canonical double molecular signature of OG, all cases in our series revealed other molecular characteristics typical of this tumor. All case showed retained nuclear expression of ATRX [23] and lacked p53 staining, consistent with the mutual exclusivity of TP53 mutation and 1p/19q deletion [24]. The majority of these cases (76%) also expressed INA which was expected since this protein is well known to be correlated to 1p/19q codeletion in the literature [8,15] and suggests a capacity for neuronal differentiation [25]. Unlike other studies in the literature [15,26], INA expression in our series was not correlated with EFS and/or OS. This discordance may be explained by our smaller cohort size if not by the fact that these earlier studies predated the WHO 2016 classification and thus classified some 1p/19q non codeleted gliomas as OGs.

As expected, the FISH technique was easy to perform on all cases of our series and the automated analysis was easily and rapidly done on each case (less than 30 min / case for 12 images acquisition and analysis) without need for manual control. Chromosome arm 9p deletion is a common progression-associated alteration in several studies, found in up to 30 to 60% of high grade and/or recurrent OGs [1,9,27,28], although not all series show such frequent alteration [24]. Our present series shows no evidence of 9p deletion in the OII cohort, but presence of 9p deletion in the majority (55%) of our OIII cases, with a strong correlation between 9p deletion and histological grading shown by univariate and multivariate analysis. Previous studies have linked 9p deletion to necrosis and/or MVP in OGs [8–10] and 9p allelic loss has been linked to unfavourable outcome in a large clinical series [28]. Our data confirm the correlation of 9p deletion with necrosis but not with MVP and confirm a strong correlation with shorter EFS and OS by univariate and multivariate analysis. Unfavourable outcome secondary to 9p deletion has been attributed to the loss of the *CDKN2A* locus on chromosome 9p21 which includes the genes for p16^INK4A^ and p14^ARF^. Protein p16, also called Cyclin-Dependent Kinase Inhibitor 2A (CDKN2A), is an inhibitor of the cyclin dependent kinases CDK4 and CDK6 which phosphorylate the retinoblastoma protein pRB (the key protein control of the cell cycle restriction point in G1 phase) and allow the progression from G1 phase to S phase [29]. By inhibiting CDK4 and CDK6, p16 impedes cell cycle progression from G1 to S phase and acts as a tumor suppressor that has been implicated in the prevention of cancers, notably gliomas [30,31]. Very few recent studies in the literature have explored p16 expression by immunohistochemistry in OGs. Studies predating the WHO 2016 molecular classification reported decreased p16 expression in a significant proportion of OGs (36 to 74%) independent of the histological grading [32–34]. This lack of expression correlated with poor survival [32,33] and was also associated with a higher proliferative activity [32]. Our results are only similar to these studies for the 9p deleted OIIId cohort in which 32% of our cases lacked p16 expression, with shorter EFS and OS by univariate analysis and with a significant association to a higher MIB1 proliferative index. Four other OIIIs in our series (10%) lacked p16 expression but were not deleted for 9p, which is also in concordance with published literature [9,33] and implies alternative genetic changes at the *CDKN2A* locus such as *CDKN2A* somatic mutation [9] or epigenetic changes such as hypermethylation [35,36]. Contrary to published literature we found no lack of p16 expression in the OII cohort and a correlation between p16 overexpression and a shorter OS in both OII and OIIIw, revealing two distinct unfavorable prognostic profiles for p16 expression according to the presence or absence of 9p deletion. This unexpected finding may be due to a statistical bias secondary to the small size of our cohort, but our series offers the advantage of being well characterized and homogeneous for IDH status and 1p 19q codeletion, which is not the case for other discordant studies in the literature. We observed two distinct outcomes for OIII according to the presence or absence of 9p deletion: the population without 9p deletion having a longer EFS and OS similar to that observed for OII and the population with 9p deletion presenting a significantly shorter OS and EFS consistent with a multistep model of oncogenesis in which multiple rounds of cell division and mutation take place, leading to acquisition of new genetic abnormalities which, together with clonal selection, shape the progression of the glioma [37]. Previous analyses of IDH mutated and 1p/19q codeleted gliomas suggest that protein p16 loss is not a consequence of deletion or mutation of the *CDKN2A* gene—as found in glioblastomas [24,37]—but rather to epigenetic changes [9,35,36] or impaired protein synthesis [32,33].

Our results suggest that 9p homozygous deletion leads to decreased protein p16 expression, loss of CDK4/6 inhibition, and an increase in cell proliferation as manifest by increased MIB-1, whereas in the absence of 9p deletion unfavorable evolution of OGs does not proceed via loss of p16 expression but probably via another mechanism resulting in the nuclear accumulation of p16 protein and its failure to fulfill its role of tumor suppressor. In a series of astrocytomas, p16 overexpression has been associated with short survival by univariate analysis but no mechanism for this finding was suggested [38]. Further studies are needed to confirm our observations and to explain their mechanism. A recent review underlines the contradictory facets of p16 expression and its significance in tumor pathobiology [13]. Genetic inactivation of p16 has been found in nearly 50% of all human cancers resulting in the bypass of this critical cell cycle control mechanism; on the other hand the overexpression of p16 at both mRNA and protein levels is associated with poor prognosis in several cancers including neuroblastoma, cervical, ovarian, breast, prostate and oral neoplasms [29] suggesting that p16 overexpression is a surrogate marker for rapid cell proliferation with failure to eliminate left-over p16 between mitoses [39].

Cyclin-D1 is a protein encoded by the CCND1 gene located on chromosome arm 11q13. It functions as a regulatory subunit of cyclin-dependent kinase 4 (CDK4) or 6 (CDK6). It dimerizes with CDK4 and CDK6 to form a complex which phosphorylates and inhibits the retinoblastoma protein (pRb) and promotes passage through the G1 phase to the S phase of the cell cycle. The binding of p16 to CDK4 or CDK6 disrupts their association with Cyclin D1 and leads to nuclear accumulation of Cyclin D1 [13]. Cyclin D1 overexpression has been shown to correlate with early cancer onset and with tumor progression [40] and has been observed in a large variety of tumors including gliomas [41,42]. As expected from its role in cell proliferation, Cyclin-D1 expression increases in anaplastic gliomas [42] and especially in OGs [11,43] showing a correlation with proliferative index [42,43]. Similar results are observed in our study with a strong correlation between Cyclin D1 overexpression and both OIII histological grade and high MIB1 proliferative index by both univariate and multivariate analysis. We also demonstrate a potential prognostic value for Cyclin D1 overexpression which appears correlated to EFS and to a lesser degree to OS in our series; this has been previously reported for astrocytomas [44] but never reported to our knowledge for OGs. The prognostic value of cyclin D1 immunostaining is not only due to the strong correlation between cyclin D1 overexpression and MIB1 overexpression (which was expected giving the molecular role of cyclin D1) but also to the strong correlation with MVP and necrosis, which represent the two major histological criteria that define anaplasia in OGs [3]. The average cyclin D1 labelling index in our series was constantly higher than that of MIB1 in the large majority of our cases (44/50 = 88%) and was not significantly correlated to hyperexpression of p16, which may be due, in addition to its role in cell cycle control, to the known abundance of cyclin D1 in Oligodendrocyte Precursor Cells (OPC) [11]. These precursor cells give rise to neurons and oligodendrocytes in the developing brain and are presumed to be the origin of OGs which would explain their proneural molecular signature including positivity for INA [11,25]. Given the present state of our data we would emphasize the diagnostic value of Cyclin D1 expression as a marker of anaplasia in OGs especially in poorly-sampled cases (biopsy and/or perilesional area). The prognostic utility of Cyclin D1 expression in OGs deserves to be confirmed by other studies.

Myc (c-Myc) is a regulatory gene located on 8q24. It belongs to the Myc family of transcriptor factors which includes N-Myc and L-Myc. It codes for a transcription factor and appears involved in the transcription of a very large panel of genes estimated to up to 15% of all human genes [45]. It codes for a nuclear phosphoprotein that plays a role in cell proliferation (by upregulating cyclins and downregulating p21), cell growth (by upregulating ribosomal RNA), apoptosis (by downregulating Bcl2) and inhibiting cell differentiation [46]. Myc is a proto-oncogene implicated in many type of neoplasm including brain tumors [47]. High levels of Myc protein expression secondary to gene amplification of Myc family members has been described in medulloblastomas [48], astrocytomas [49] and glioblastomas [50]. Genotyping studies showed variants mapping to 8q24, near the c-Myc locus, associated with increased risk for development of IDH mutant gliomas [51]. It has since been demonstrated that increased Myc activity is associated with malignant progression and worse prognosis in IDH-mutated gliomas [52,53] and especially in OGs [12]. Our study confirmed the high expression of Myc in all our OG cases compared to normal brain controls. It also confirmed the link between Myc overexpression and progression to anaplasia with a significant correlation with histological grading but not with proliferative index, MVP or necrosis as seen with Cyclin D1 and/or p16. Myc overexpression appeared only correlated to p16 overexpression which was unexpected giving the presumed inhibitory role of Myc on p16 function [14,46]. These data underline the complexity of molecular interactions in tumor process and may indicate an escape of p16 from Myc inhibitory control in OGs, but giving the absence of specific information on this subject in the literature, these findings need to be confirmed in future studies. In our study, the only significant prognostic impact of Myc expression was on EFS in the OIIIw subgroup. We would have expected an impact on OIIId EFS as observed by others [12]. This discrepancy is worth investigating further to see if it may be explained by differences in methodology, since other factors such as miRNA levels and post-translational modifications affecting protein turnover could affect the correlation between genomic expression data on one hand and protein levels on the other.

Recent molecular and epigenetic studies of OGs have identified a poor prognosis subgroup of anaplastic OG characterized by the presence of vascular proliferation, necrosis, 9p deletion and high Myc activity [12]. This subgroup presents a higher expression of oligodendrocyte precursor cell (OPC) markers, especially GPR17 an oligodendrocyte-specific G-protein-coupled receptor [54] and cyclin D1 [11]. In our study we confirm the presence of a similar poor prognosis subgroup of anaplastic OG characterized by 9p deletion, necrosis, high proliferative and mitotic index and loss of p16 expression. In this subgroup we also found Cyclin D1 and Myc overexpression but these expression levels were not significantly different from those in the rest of the cohort and there was no significant correlation with EFS and OS. In our study the prognostic impact of Myc overexpression in OG does not appear as clearly established that of 9p deletion or loss of p16 expression and deserves to be clarified in further studies.

## Conclusions

This study confirms the reliability of automated 9p FISH analysis and its value in assessing the prognosis of anaplastic OGs. 9p deletion appeared strongly correlated with high grade OG status and with poor EFS and OS by univariate and multivariate analysis. Further stratification of OGs can be achieved by immunohistochemical studies available in most anatomic pathology laboratories. Analysis of p16 expression permits identification of a subgroup of OIII characterized by 9p homozygous deletion and p16 lack of expression with a particularly poor EFS and OS. Such patients would likely benefit from closer clinical follow up and might require a more intensive or a different chemotherapy protocol than usual, since OGs are usually chemosensitive. Cyclin D1 overexpression appeared to be a good marker of progression towards anaplasia in OGs and might be of prognostic interest if these findings are confirmed in further studies. Myc overexpression increased in parallel with the degree of malignancy in OGs, but showed no clear correlation with survival.

## Supporting information

S1 File Research ethics committee opinion. (PDF)
