# Supplementary material for: Impact of 9p deletion and p16, Cyclin D1, and Myc hyperexpression on the outcome of anaplastic oligodendrogliomas
Source: PLoS One. 2018 Feb 28;13(2):e0193213. doi: 10.1371/journal.pone.0193213 (PMC5831111; doi:10.1371/journal.pone.0193213)
Supplement: S1 File — (PDF) [file pone.0193213.s001.pdf]

CHU de Québec Université Laval

Par courrier électronique seulement

Le 12 janvier 2017

Docteur Stéphan Saikali
Neuropathologie
Hôpital de l'Enfant-Jésus
CHU de Québec-Université Laval

Objet : Réponse à la demande d'avis 2017-3456/ Assurance qualité, mandat de l'organisation
Étude de l'hyper-expression des protéines cycline D1 et cMYC dans les oligodendrogliomes et de leur corrélation avec la délétion 9p : facteur diagnostic et pronostique

---

Docteur,

Votre demande 2017-3456 concernant la nécessité ou non d'obtenir une approbation du Comité d'éthique de la recherche du CHU de Québec – Université Laval a été évaluée. Après l'avoir analysé, il appert que, selon l'Énoncé de politique des trois Conseils, *Éthique de la recherche avec des êtres humains, 2014 (EPTC2, article 2,5),* votre devis de recherche n'est pas considéré comme un projet de recherche nécessitant une approbation par un comité d'éthique de la recherche.

Conséquemment le CÉR du CHU de Québec – Université Laval n'a pas à approuver votre projet afin qu'il puisse se dérouler au sein de l'établissement. Toutefois, les considérations habituelles pour l'accès aux dossiers hospitaliers par le Directeur des services professionnels du CHU de Québec – Université Laval et le respect de la confidentialité selon les règles des Bonnes Pratiques Cliniques sont applicables.

Les informations recueillies ne devront servir qu'aux seuls objectifs de la présente activité d'évaluation et ne devront pas faire l'objet d'une banque de données ou être utilisées ultérieurement à d'autres fins Il est recommandé de procéder à l'anonymisation irréversible une fois que les analyses sont complétées afin de permettre une protection maximale de la confidentialité.

Je vous prie d'agréer, Docteur, l'expression de mes sentiments les meilleurs.

Me Edith Deleury
Présidente-coordonnatrice
Comité d'éthique de la recherche
CHU de Québec – Université Laval

ED/mnl
